# Virtual Screening of Marine Natural Compounds by Means of Chemoinformatics and CDFT-Based Computational Peptidology

**DOI:** 10.3390/md18090478

**Published:** 2020-09-20

**Authors:** Norma Flores-Holguín, Juan Frau, Daniel Glossman-Mitnik

**Affiliations:** 1 Laboratorio Virtual NANOCOSMOS, Departamento de Medio Ambiente y Energía, Centro de Investigación en Materiales Avanzados, Chihuahua, Chih 31136, Mexico; norma.flores@cimav.edu.mx; 2 Departament de Química, Universitat de les Illes Balears, E-07122 Palma de Malllorca, Spain; juan.frau@uib.es

**Keywords:** theopapuamides A-D, virtual screening, chemoinformatics, conceptual DFT, computational peptidology, bioavailability, bioactivity scores, ADME

## Abstract

This work presents the results of a computational study of the chemical reactivity and bioactivity properties of the members of the theopapuamides A-D family of marine peptides by making use of our proposed methodology named Computational Peptidology (CP) that has been successfully considered in previous studies of this kind of molecular system. CP allows for the determination of the global and local descriptors that come from Conceptual Density Functional Theory (CDFT) that can give an idea about the chemical reactivity properties of the marine natural products under study, which are expected to be related to their bioactivity. At the same time, the validity of the procedure based on the adoption of the KID (Koopmans In DFT) technique, as well as the MN12SX/Def2TZVP/H_2_O model chemistry is successfully verified. Together with several chemoinformatic tools that can be used to improve the process of virtual screening, some additional properties of these marine peptides are identified related to their ability to behave as useful drugs. With the further objective of analyzing their bioactivity, some useful parameters for future QSAR studies, their predicted biological targets, and the ADMET (Absorption, Distribution, Metabolism, Excretion and Toxicity) parameters related to the theopapuamides A-D pharmacokinetics are also reported.

## 1. Introduction

Drug design can be pursued following a methodology driven by advancement and innovation breakthroughs including a combination of experimental and computational strategies. Computational strategies play a pivotal part in advanced therapeutic chemistry, displaying a special potential for changing the early stages in the process of drug discovery, especially in terms of time and cost savings [1,2].

Bioactive peptides are short amino acid chains that are inactive within the sequence of the parent protein and can be activated through gastrointestinal digestion, food processing, storage, or in vitro hydrolysis by proteolytic enzymes. Since bioactive peptides have the ability to impart a biological effect resulting in a positive impact on body functions or conditions, their application is of relevance for the food industry. Computational chemistry and molecular modeling tools provide methodologies to identify peptide sequences and their 3D molecular structure. Using in silico modeling, these molecular structures can be related to biological activity and targets of interest. These in silico targeted discovery approaches are well known and frequently used in drug discovery in the pharmaceutical industry. Computational chemistry methodologies are based on a broad palette of experience in pharmaceutical drug discovery, and the application of this knowledge towards medicinal chemistry research may lead to new insights through the elucidation of the mechanistic knowledge of potential ingredients such as bioactive peptides.

Since the structure of molecules defines the chemical, physical, and biological properties of matter, this information is crucial for understanding, explaining, and predicting chemical reactions and biochemical processes, developing new drugs, and providing crucial insights into the nature of the interactions between drug targets and ligands, which allows predictive models that are suitable for lead discovery and optimization to be constructed. There are numerous reports in the literature showing that from a molecular viewpoint, the bioactivity and the chemical reactivity properties of these peptides are intimately related [3,4]. For this reason, we are currently doing exhaustive research in this field by studying different families of marine peptides (mainly cyclodepsipeptides), trying to find the relationships that could help in the development of new medical drugs to fight several diseases.

It then follows that it is of utmost importance to study the chemical reactivity of natural products (in this case, of marine origin), because this is likely to help with the development of some medicines with the aid of tools displayed through computational and theoretical chemistry, as well as molecular modeling. In a very special way, Conceptual Density Functional Theory (DFT) [5,6,7] is one of the most powerful tools among those that are available for the study, understanding, and comprehension of the chemical reactivity of molecular systems. Conceptual DFT, sometimes called chemical reactivity theory, is able to help in the prediction of the relationships between the bioactivity and the chemical reactivity properties by considering a series of global and local descriptors that arise from the fundamentals of the method [8,9,10].

**Figure 1:**
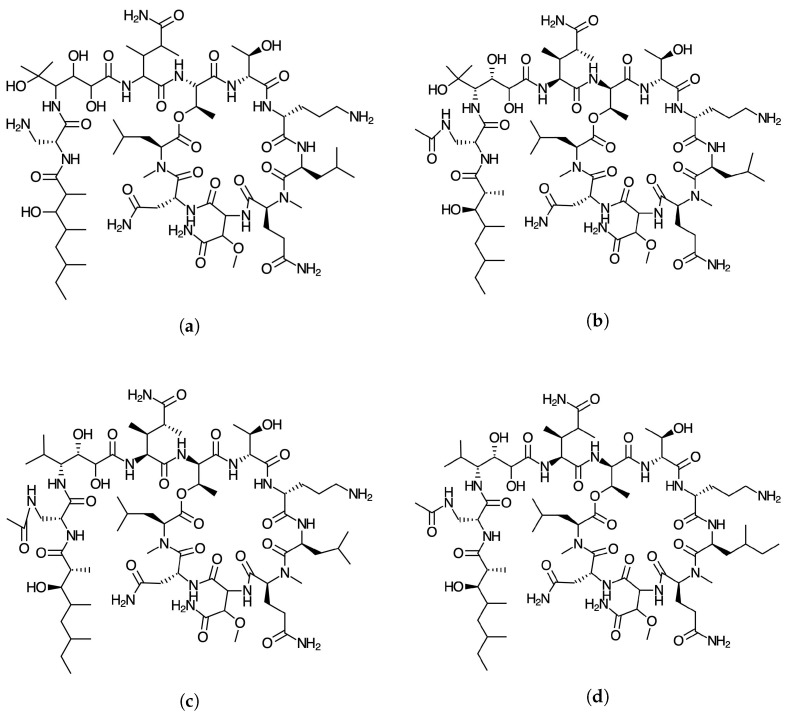
Graphical sketches of the molecular structure of (**a**) theopapuamide A, (**b**) theopapuamide B, (**c**) theopapuamide C, and (**d**) theopapuamide D.

As a part of an ongoing project for the development of new pharmaceutical drugs of marine origin, we are currently investigating new families of peptides obtained from marine sources, hoping that this could be the starting point for the design of potentially helpful therapeutic peptides [11]. Thus, the objective of this study is to report the global and local chemical reactivity descriptors of the theopapuamides A-D family of marine peptides [12,13,14,15], whose graphical sketches are shown in Figure 1 by making use of the Conceptual DFT methodology. It also involves the determination of the potential reaction sites for the cases of nucleophilic and electrophilic attacks, and though the consideration of a methodology that we have developed and validated before, also the prediction of the pKa values for each peptide has been attained [16]. The study has been complemented by considering the report of some additional properties that could be useful in QSAR involving several methodologies addressed in the literature [17,18], as well as a detailed study of bioactivity radar charts that can give an idea of the drug-like behavior of the studied peptides, the predicted biochemical targets on the basis of a homology methodology, and the values associated with the pharmacokinetics. By following this approach that we have coined Conceptual DFT-based Computational Peptidology, as a branch of computational chemistry dedicated to the study of peptides, the current study acts as a follow-up to previously published results on some families of therapeutic peptides of marine origin [19,20,21,22,23].

## 2. Theoretical Background and Computational Details

The Kohn–Sham (KS) methodology includes the estimation of the molecular energy and density of a given system, as well as the orbital energies, explicitly connected with the frontier orbitals including the Highest Occupied Molecular Orbital (HOMO) and Lowest Unoccupied Molecular Orbital (LUMO) [24,25,26,27]. This methodology is convenient when thinking of quantitative qualities related to Conceptual DFT descriptors. At present, the utilization of Range-Separated (RSE) exchange-correlation density functionals in Kohn–Sham DFT is of extraordinary concern [28,29,30,31]. It is essential to the development of these density functionals to think about the partitioning of the exchange and the $r_{12}^{-1}$ operator into long-and short-range parts partnering with a range-separation ω parameter that controls the rate at which long-range behavior is obtained. The estimation of ω can either be fixed or “tuned” by the utilization of a molecule-by-molecule procedure by adhering to some tuning principles. The ideal tuning methodology depends on having the KS HOMO energy related to the vertical Ionization Potential (IP), which is an estimation of the energy difference, E(N−1) − E(N). For the generalized KS theory appropriate for an N-electron molecular system, we should have -IP(N) = $\epsilon_{H}$(N), which can be considered as the DFT counterpart of the well-known Koopmans’ theorem. In reality, this is valid only for the exact density functional. For the situation where, for pragmatic reasons, we have to consider an approximated density functional, and there will possibly be some critical distinction between -IP(N) and $\epsilon_{H}$(N). Thus, ideal tuning involves setting up ω, the system-specific range separation parameter, by a nonempirical approach and having an RSE useful density functional [32,33,34,35,36,37,38,39]. Indeed, even with the absence of an equal methodology that can be utilized for the correlation of the Electron Affinity (EA) combined with the energy of the LUMO, it can be shown that $\epsilon_{H}$(N + 1) = -EA(N) is conceivable. This makes the acquisition of the optimized ω value easier, provided that the differences between $\epsilon_{L}$(N) and $\epsilon_{H}$(N + 1) are small. Through this, the forecast of the Conceptual DFT descriptors’ expectation is upgraded for a given optimized density functional. The concurrent prescription is dubbed the “KID procedure” (for Koopmans In DFT), this being in reference to the relationship it has with Koopmans’ theorem that has been previously quoted [19,20,21,22,23].

Following the methodology considered in our previous studies [19,20,21,22,23], we have done the computational determinations by using the Gaussian 09 series of programs [40] for the implementation of the density functional needed for the development of this work. The Def2SVP basis set [41,42] was chosen for the geometry optimizations and in the verification that the optimized structures corresponded to the minimal ones through the calculation of the associated frequencies. As a larger basis set is usually needed for the calculation and analysis of the electronic properties, the Def2TZVP basis set was chosen [41,42] as a constituent of our standard methodology. According to our previous studies on this kind of molecular systems, water was selected as the solvent through the Solvation Model Density (SMD) parameterization of the Integral Equation Formalism-Polarized Continuum Model (IEF-PCM) [43] for all the DFT calculations. For the determination of the molecular structures and the associated electronic properties of the studied peptides, the MN12SX density functional was chosen because it is already well known that it is capable of giving very good results for several structural and thermodynamic properties [44]. The resulting model chemistry, MN12SX/Def2TZVP/H2O, has proven to be adequate because MN12SX behaves as a Koopmans-compliant density functional, which is a very helpful feature for obtaining accurate HOMO and LUMO energies, avoiding the determination of the energies of the cationic and anionic systems for which convergence is usually hard to obtain for somewhat large molecules, as peptides are [19,20,21,22,23].

## 3. Results and Discussion

The starting molecular structures of the peptides theopapuamides A-D to be studied were obtained from PubChem (https://pubchem.ncbi.nlm.nih.gov), which is an open chemistry database that acts as an online free resource of information related to physical, chemical, and biological properties, interactive spectra, and literature references. In order to get a glimpse of the potential therapeutic properties of the considered peptides, Simplified Molecular Input Line Entry Specification (SMILES) notations for the molecular systems under consideration were fed into the online Molinspiration software from Molinspiration Cheminformatics (Slovensky Grob, Slovak Republic) allowing for the estimation of several molecular properties that are known to be related to drugability and that could be useful in QSAR studies that are commonplace in the process of drug design and development. The results of this determination are presented in Table 1.

The values of the pKas of the theopapuamides A-D displayed in Table 1 were calculated by resorting to a methodology proposed by our research group [16], which has been proven to be successful for this task in the study of several families of peptides of marine origin [19,20,21,22,23].

**Figure 2:**
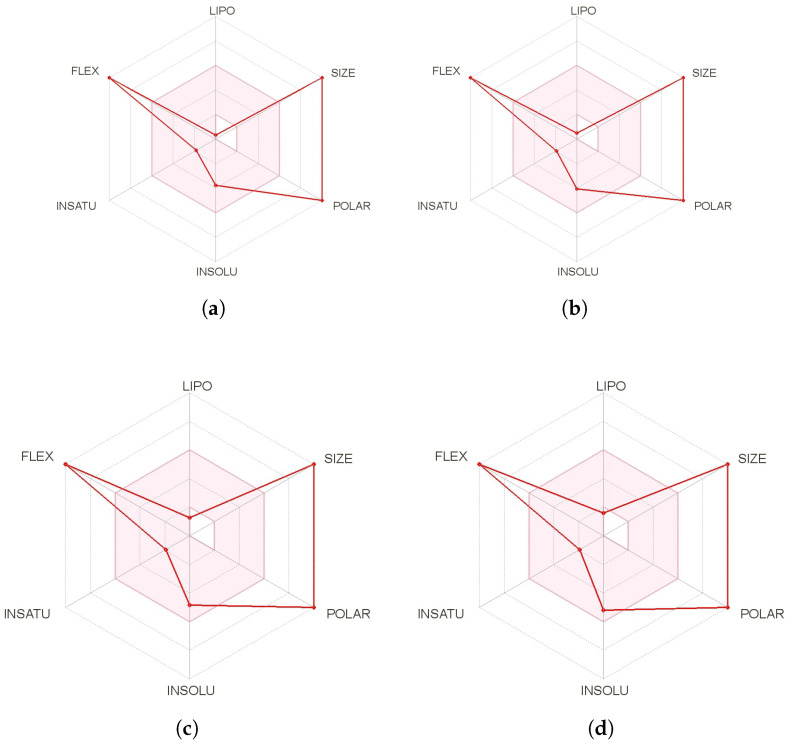
Bioactivity radar charts of the (**a**) theopapuamide A, (**b**) theopapuamide B, (**c**) theopapuamide C, and (**d**) theopapuamide D molecules where FLEX: Flexibility, LIPO: Lipophilicity, INSATU: Insaturation and INSOLU: Insolubility.

A further and complementary step can be performed by resorting to SwissADME [45], which is a free online tool that allows the evaluation of drug-likeness though a graphical representation of the properties of interest called the bioavailability radar chart, which is obtained for every peptide with the aid of its SMILES representation and where the pink area exhibits the zone with the optimal range for a particular property, as shown in Figure 2.

It can be appreciated from Figure 2 that the major drawbacks for these peptides to be considered as potential drugs are related to their large size and flexibility. These are very common features of peptides that could prevent their consideration as therapeutic drugs. However, these problems could be overcome by considering peptidomimetics studies and finding alternative ways to facilitate the drug delivery process.

Another piece of information that can be obtained from the precedent study is that related to the pharmacokinetic properties of the potential therapeutic peptides, that is how the living organism will interact with the drugs since they are delivered to the body up to when they or their metabolites are finally excreted. This information is collectively known as the ADME properties, and the experimental evaluation of ADME is still costly and time consuming. Thus, the development of computer science and modeling has become a useful tool to predict ADME properties, and this important information in the process of drug discovery, which has been also obtained with the aid of SwissADME [45], is presented in Table 2 for the marine peptides theopapuamides A-D.

**Figure 3:**
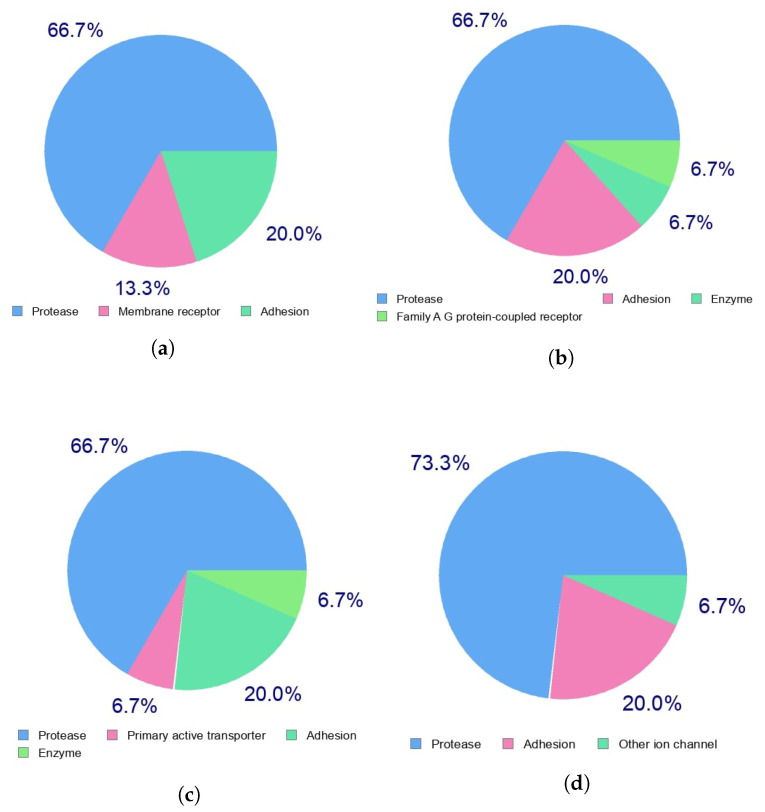
Predicted biological targets of the (**a**) theopapuamide A, (**b**) theopapuamide B, (**c**) theopapuamide C, and (**d**) theopapuamide D molecules.

Indeed, the efficacy of a given therapeutic drug will be highly dependent of the way it interacts with a given receptor in the cells and, in turn, with the diseases that the medicines will be designed to fight. This can be predicted beforehand by resorting to a homology procedure using databases of known molecules of similar and related structures to the ones under study. This is an important tool in the drug discovery process and has been used in this work to analyze the theopapuamides A-D family of peptides by resorting to the free online SwissTargetPrediction software [46] with the results for the predicted biological targets presented in Figure 3.

All the presented results for the potential therapeutic activity of the theopapuamides A-D family of peptides are an indication that the chemical reactivity of these molecules is an area worth exploring. As we did in the past [19,20,21,22,23], a methodology called Conceptual DFT-based Computational Peptidology developed in our research group will be considered now for the calculation and analysis of the chemical reactivity properties of these interesting molecules. The starting point consists of a search for the most stable conformers of each peptide on the basis of the molecular structures taken from the online chemistry database PubChem by resorting to the MarvinView 17.15 program (ChemAxon, Budapest, Hungary) relying on the overall MMFF94 force field [47,48,49,50,51].

The most stable conformers for each peptide obtained through the described procedure were then subjected to a geometry optimization in the gas phase by considering the Density Functional Tight-Binding Approximation (DFTBA) model that is accessible within Gaussian 09 [40]. As was mentioned in the Theoretical Background and Computational Details section, the resultant structures were subsequently reoptimized by considering the MN12SX/Def2SVP/H2O model chemistry, which has proven to be adequate for this purpose [19,20,21,22,23]. Upon verification that every optimized structure corresponded to a minimum in the energy potential curve by employing the frequency-calculation analysis technique, the electronic properties were calculated by resorting to a similar model chemistry, but considering the Def2TZVP basis set instead of the Def2SVP one because it has been demonstrated [19,20,21,22,23] that this is a better choice for the prediction and analysis of the HOMO and LUMO and of the chemical reactivity properties derived from them.

**Figure 4:**
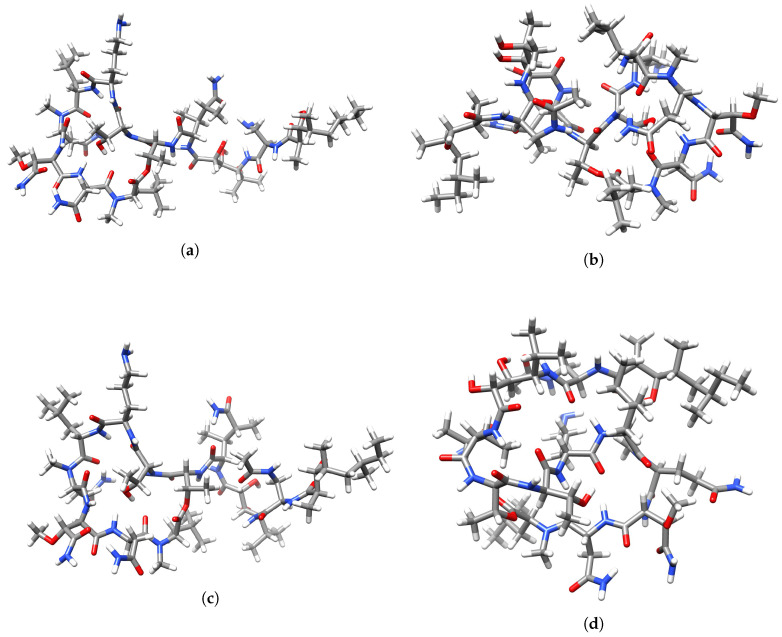
Optimizedmolecular structures of (**a**) theopapuamideA, (**b**) theopapuamide B, (**c**) theopapuamide C, and (**d**) theopapuamide D.

A graphical display of the tridimensional optimized molecular structures of the peptides theopapuamides A-D obtained with the aid of the UCSF Chimera Visualization System [52] are presented in Figure 4.

It is usually assumed that the goodness of a given density functional can be estimated by comparing the results that it gives with the experimental values that are attempted to be reproduced or with the results that can be obtained through post-Hartree–Fock calculations like MP2, MP4, or CCSD. However, this is not always possible due to the lack of experimental results for the molecular systems that are being studied or the large size of the molecules that prevent some accurate methodologies from being computationally practical. For this reason, we developed a protocol named KID (Koopmans In DFT) [19,20,21,22,23], which is an attempt to validate a given density functional in terms of its internal coherence. Within the KID protocol, four descriptors have been defined, where it has been shown that there is a connection between those descriptors and the simplest conformity to the theorem of Koopmans or the ionization energy theorem, which is its equivalent within the Generalized Kohn–Sham (GKS) version of DFT, by connecting $\epsilon_{H}$ to -I, $\epsilon_{L}$ to -A, and their actions through the HOMO–LUMO gap as $J_{I}=\left|\epsilon_{H}+E_{gs}\left(N-1\right)-E_{gs}\left(N\right)\right|$, $J_{A}=\left|\epsilon_{L}+E_{gs}\left(N\right)-E_{gs}\left(N+1\right)\right|$, and $J_{HL}=\sqrt{{J_{I}}^{2}+{J_{A}}^{2}}$. An additional descriptor ΔSL has been designed [19,20,21,22,23] to help in the verification of the accuracy of the KID approximation by comparing the HOMO energy of the radical anion (or SOMO) with the energy of the LUMO of the neutral species. Although the Koopmans-compliant behavior of the MN12SX density functional has been proven previously for the case of peptides [19,20,21,22,23], we think that it is worth performing a further validation for the case of the molecules considered in the present study. This determination was achieved by making use of the in-house developed CDFT software tool, and the results of this analysis are shown in Table 3.

As can be seen from the results in Table 3, the values for the KID descriptors are all very close to zero, which is an indication that the chosen MN12SX density functional behaves as a Koopmans-compliant one and that for this reason, the MN12SX/Def2TZVP/H2O is a model chemistry that has been further demonstrated to be very adequate for the purpose of this research.

Taking into account the KID methodology considered in the previous research being integrated into the finite difference approximation [19,20,21,22,23], the following definitions can be used for the global descriptors that help in the understanding of the chemical reactivity of the molecular systems [5,6,7,53,54]:
Electronegativity$\chi =-\frac{1}{2}(I+\left(a\right)\approx \frac{1}{2}\left(\epsilon_{L}+\epsilon_{H}\right)$
Global hardness$\eta =(I-\left(a\right)\approx \left(\epsilon_{L}-\epsilon_{H}\right)$
Electrophilicityω = $\frac{\mu^{2}}{2\eta }=\frac{(I+{\left(a\right)}^{2}}{4(I-(a)}\approx \frac{{\left(\epsilon_{L}+\epsilon_{H}\right)}^{2}}{4(\epsilon_{L}-\epsilon_{H})}$
Electrodonating power$\omega^{-}$ = $\frac{(3I+{\left(a\right)}^{2}}{16(I-(a)}\approx \frac{{\left(3\epsilon_{H}+\epsilon_{L}\right)}^{2}}{16\eta }$
Electroaccepting power$\omega^{+}$ = $\frac{(I+3{\left(a\right)}^{2}}{16(I-(a)}\approx \frac{{\left(\epsilon_{H}+3\epsilon_{L}\right)}^{2}}{16\eta }$
Net electrophilicity$\Delta \omega^{\pm }=\omega^{+}-\left(-\omega^{-}\right)=\omega^{+}+\omega^{-}$

$\epsilon_{H}$ and $\epsilon_{L}$ being the HOMO and LUMO energies associated with each of the peptides considered in this work.

As a complement of these global reactivity descriptors that arise from Conceptual DFT [5,6,7,53,54], Domingo and his collaborators [55,56,57,58,59] proposed a Nucleophilicity index (N) through the consideration of the HOMO energy obtained through the KS scheme with an arbitrary shift of the origin, taking the molecule of Tetracyanoethylene (TCE) as a reference.

By making use of the mentioned CDFT software tool applied to the results of the calculation of the electronic properties of the peptides theopapuamides A-D, the values of the defined global reactivity descriptors (including the Nucleophilicity (N)) can be obtained, and they are displayed in Table 4.

The global hardness η can be seen as a measure of the resistance of the electronic density to being deformed and thus as an indication of the low reactivity of a given molecular system. From the results of Table 4, it can be concluded that theopapuamide B will be the more reactive peptide in this family, while theopapuamide D will be the less reactive one, the chemical reactivity of the other peptides being approximately the same. An analogous behavior is observed for the electrophilicity ω descriptor, which encompasses the balance between the tendency of an electrophile to acquire an extra amount of electrons and the resistance of a molecule to exchange electrons with the environment. As expected from the molecular structure of these species, their electrodonating ability is more important than their electroaccepting character, theopapuamides B and D possessing an electrodonating power that is greater than the others, but not very different. On the basis of the previous definition and the scale established by these authors [56], theopapuamide D can be regarded as a strong nucleophile because the values for the Nucleophilicity (N) are greater than 3 eV, while the other peptides can be considered as moderate nucleophiles.

The presented global descriptors are a representation of the chemical reactivity of a molecule as a whole. However, local reactivity descriptors have been developed that can give an idea about the differences between the reactivity of each of the atoms that form the molecule. One of the most important groups of such reactivity descriptors are the Fukui functions [5,6,7] and the Dual Descriptor [60,61,62,63,64,65], which is defined as:
Nucleophilic Fukui function$f^{+}\left(r\right)=\rho_{N+1}\left(r\right)-\rho_{N}\left(r\right)$
Electrophilic Fukui function$f^{-}\left(r\right)=\rho_{N}\left(r\right)-\rho_{N-1}\left(r\right)$
Dual descriptorΔf(r) = ${\left(\frac{\partial \;f(r)}{\partial \;N}\right)}_{\upsilon (r)}$

which are the relationships between the electron densities of the neutral, positive, and negative species, as well as between the nucleophilic and electrophilic Fukui functions.

**Figure 5:**
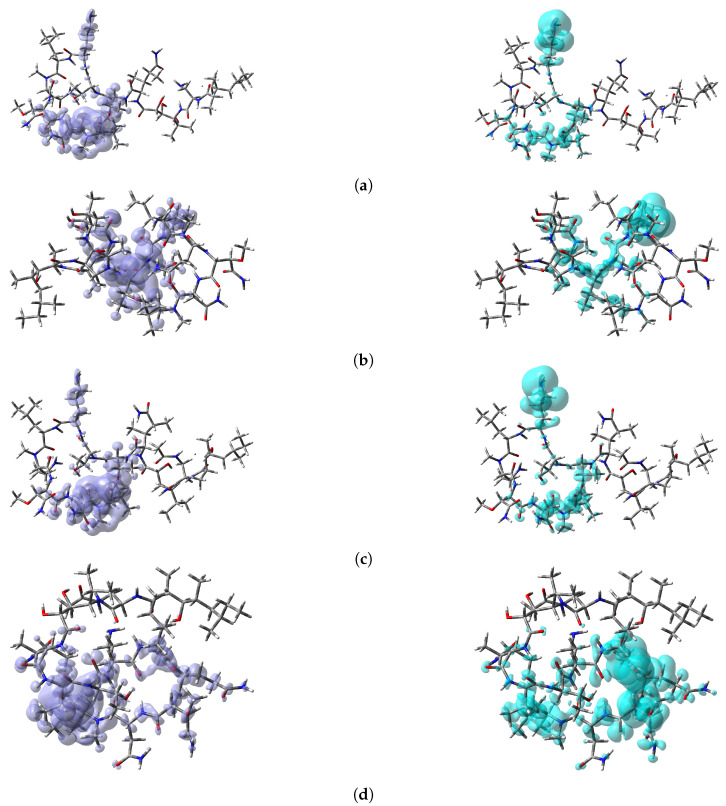
Graphical representation of the Dual Descriptor (DD) of the (**a**) theopapuamide A, (**b**) theopapuamide B, (**c**) theopapuamide C, and (**d**) theopapuamide D molecules. Left: DD > 0; right: DD < 0.

The nucleophilic Fukui function, $f^{+}\left(r\right)$, reveals the sites on a molecular system that are susceptible to nucleophilic attack, and the electrophilic Fukui function, $f^{+}\left(r\right)$, describes those sites that are more susceptible to electrophilic attack. These local reactivity descriptors are very useful and have been used successfully for the identification of reactive sites. However, sometimes, there is an overlap between the results of both descriptors, and no conclusions can be accurately obtained. Instead, the Dual Descriptor (DD) Δf(r) can unambiguously describe the nucleophilic and electrophilic sites within a molecule [65]. Thus, a graphical representation of the DD for the marine theopapuamide peptides is presented in Figure 5, clearly showing the areas within the molecules where DD > 0 and DD < 0 for a better understanding of the local chemical reactivity of these molecules.

Another local reactivity descriptor is the Parr functions [66,67], which can be considered as an alternative to the Fukui functions to describing the sites or areas within the molecules where nucleophilic or electrophilic attack will be favored. The Parr functions can be expressed as [66,67]:
Nucleophilic Parr function$P{}^{-}\left(r\right)=\rho_{s}^{rc}\left(r\right)$
Electrophilic Parr function$P{}^{+}\left(r\right)=\rho_{s}^{ra}\left(r\right)$
where $\rho_{s}^{rc}\left(r\right)$ and $\rho_{s}^{ra}(r$) are related to the atomic spin density of the radical cation or anion of the considered system, respectively [59].

In order to perform a comparison between the results that can be obtained from the electrophilic and nucleophilic Fukui function analysis for each peptide and the values that are the outcome of the Parr function analysis, the predictions for the specific reactions sites are compiled in a series of tables that are presented as the Appendix A to this work. The radical Fukui function $f^{0}\left(r\right)$, which can be considered as an average of $f^{+}\left(r\right)$ and $f^{-}\left(r\right)$ and which denotes the favorable sites for radical attack, is also included for the sake of completeness. In a complementary way to the tables, a graphical representation of these descriptors is included as a series of figures, so that the comparison between the results coming for both kinds of studies can be done accurately. Although partial charges for the molecules could be obtained after the energy minimization procedure, they were actually extracted from the corresponding molecular wavefunctions using the the Multiwfn 3.7 program [68] during the determination of the Fukui functions.

**Figure A1:**
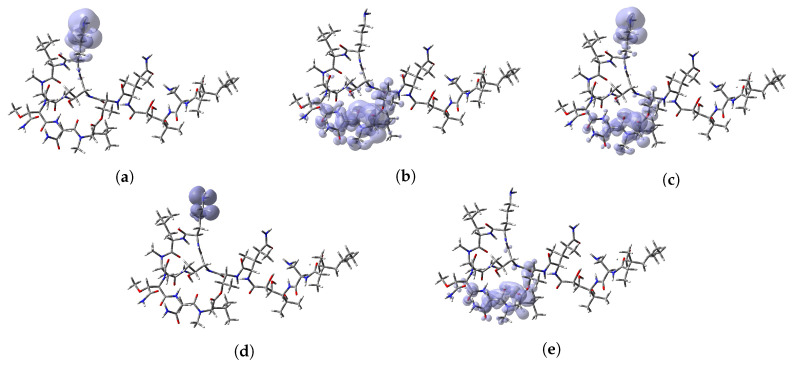
Graphical sketches of the local reactivity descriptors for theopapuamide A showing the reactivity regions: (**a**) electrophilic Fukui function $f^{-}$, (**b**) nucleophilic Fukui function $f^{+}$, (**c**) radical Fukui function $f^{0}$, (**d**) electrophilic Parr function P−, and (**e**) nucleophilic Parr function P+.

**Figure A2:**
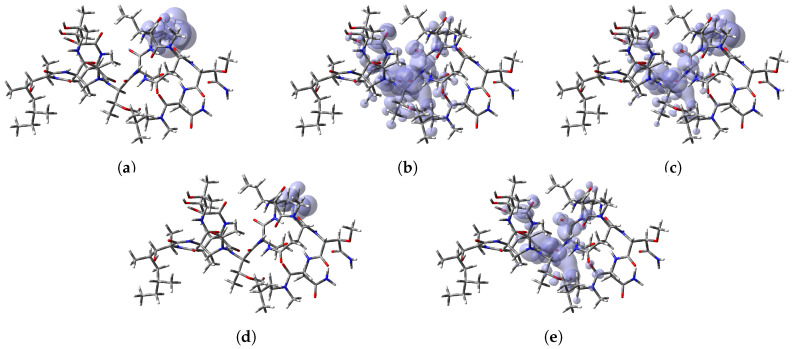
Graphical sketches of the local reactivity descriptors for theopapuamide B showing the reactivity regions: (**a**) electrophilic Fukui function $f^{-}$, (**b**) nucleophilic Fukui function $f^{+}$, (**c**) radical Fukui function $f^{0}$, (**d**) electrophilic Parr function P−, and (**e**) nucleophilic Parr function P+.

**Figure A3:**
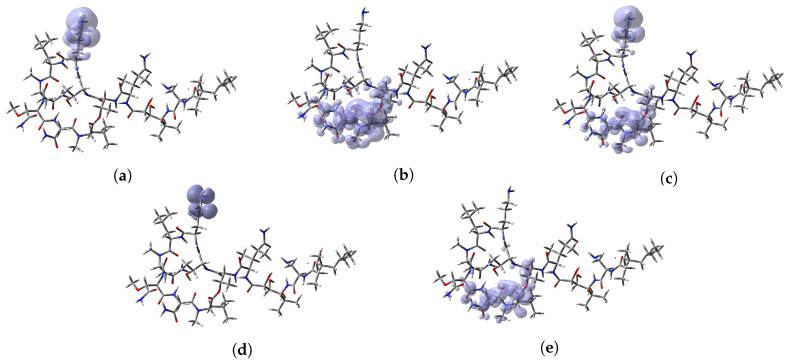
Graphical sketches of the local reactivity descriptors for theopapuamide C showing the reactivity regions: (**a**) electrophilic Fukui function $f^{-}$, (**b**) nucleophilic Fukui function $f^{+}$, (**c**) radical Fukui function $f^{0}$, (**d**) electrophilic Parr function P−, and (**e**) nucleophilic Parr function P+.

**Figure A4:**
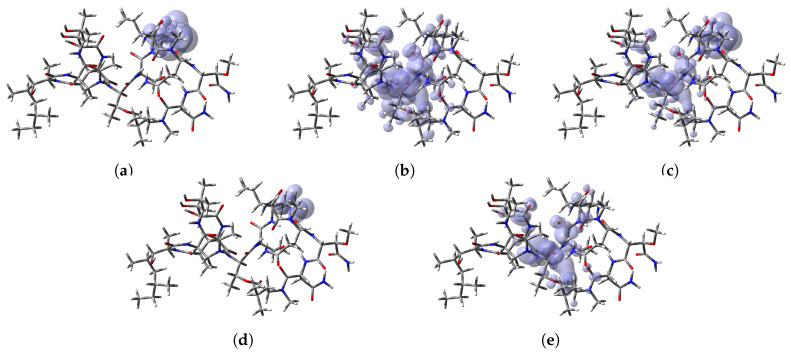
Graphical sketches of the local reactivity descriptors for theopapuamide D showing the reactivity regions: (**a**) electrophilic Fukui function $f^{-}$, (**b**) nucleophilic Fukui function $f^{+}$, (**c**) radical Fukui function $f^{0}$, (**d**) electrophilic Parr function P−, and (**e**) nucleophilic Parr function P+.

By looking at the numerical and graphical results presented in Table A1, Table A2, Table A3 and Table A4 and Figure A1, Figure A2, Figure A3 and Figure A4, it can be concluded that there is a very nice agreement between the values coming from both representations. Considering the numerical values, it can be seen that there is a complete agreement between the reactive sites predicted by the Fukui functions or the Parr functions. It can also be noted that the numerical results for the Parr functions are greater than those for the Fukui functions, which is an indication that the first ones are better defined than the others. The same conclusions can be obtained by looking at the graphical representations of the Fukui and Parr functions, where they are extended over the same surface within the molecules, but it can be seen that the Parr functions are more compact than the Fukui functions. This nice agreement, which has also been found in the study of other families of peptides of marine origin, could lead to the conclusion that if the DD can be built as the difference between both Fukui functions, a new reactivity descriptor could be defined in terms of the difference between both Parr functions in an analogous way to the dual descriptor giving the same information and being the starting point for further research in the field of Conceptual DFT-based Computational Peptidology.

## 4. Conclusions

The theopapuamides A-D family of cyclodepsipeptides of marine origin are studied by resorting to some techniques of common use in the process of drug discovery and development, showing that these kinds of molecules can be regarded as potential therapeutic drugs. Some chemoinformatic tools are used to obtain information about the potential therapeutic properties of these peptides in the form of bioactivity radar charts, biological targets, and ADME values.

With this knowledge in mind, the chemical reactivity of the studied peptides is exhaustively analyzed through the optimization of their structures using an MN12SX/Def2SVP/H2O model chemistry and the determination of their electronic properties by means of a larger model chemistry, namely MN12SX/Def2TZVP/H2O, already used in previous works for the study of peptides, validating their usefulness for these kinds of calculations.

The chemical reactivity of the considered molecular systems is subjected to an analysis based on a particular methodology developed by our research group named Conceptual DFT-based Computational Peptidology, the use of the MN12SX density functional being validated once again by resorting to the KID procedure and the in-house software tool CDFT.

The analysis of the global and local reactivity descriptors arising from Conceptual DFT together with some proposals like the Nucleophilicity (N) and the Parr functions allows for a complete understanding of the chemical reactivity of the studied peptides, by distinguishing the different chemical reactivities through the analysis of the global descriptors and further identification of the reaction sites or regions within the molecules by resorting to Fukui and Parr functions, as well as the dual descriptor.

Finally, a nice agreement is found between the outcome of the numerical and graphical analysis of the Fukui and Parr functions on each peptide, leading to the conclusion that it could be possible to define a new chemical reactivity descriptor analogous to the dual descriptor on the basis of the difference between the Parr functions, which could act as an additional tool for the study of the chemical reactivity of molecular systems through Conceptual DFT-based Computational Peptidology.

## Figures and Tables

**Table 1:** Predicted parameters useful for QSAR studies for the marine cyclopeptides theopapuamides A-D: ΔG of solvation (in Kcal/mol), pKa, logP, TPSA(Å_2_), and molecular volume (Å_3_).

	ΔG of Solvation	pKa	logP	TPSA	Molecular Volume
Theopapuamide A	−92.97	11.77	−5.58	663.60	1455.23
Theopapuamide B	−91.59	11.99	−5.50	666.78	1491.89
Theopapuamide C	−93.67	11.82	−5.27	646.45	1484.20
Theopapuamide D	−87.85	11.54	−5.20	646.45	1501.00

**Table 2:** Absorption, Distribution, Metabolism, and Excretion (ADME) parameters related to the theopapuamides A-D pharmacokinetics.

	Theopapuamides			
	A	B	C	D
GI absorption	Low	Low	Low	Low
BBB permeable	No	No	No	No
P-gp substrate	Yes	Yes	Yes	Yes
CYP1A2 inhibitor	No	No	No	No
CYP2C19 inhibitor	No	No	No	No
CYP2C9 inhibitor	No	No	No	No
CYP2D6 inhibitor	No	No	No	No
CYP3A4 inhibitor	No	No	No	No
Log K${}_{p}$ (skin permeation)	−19.30	−19.37	−18.28	−17.98
(cm/s)				

**Table 3:** HOMO, LUMO, and SOMO orbital energies, HOMO-LUMO gap, and Koopmans In DFT (KID) descriptors (all in eV) tested in the verification of the Koopmans-like behavior of the MN12SX density functional for the marine cyclopeptides theopapuamides A-D.

	HOMO	LUMO	SOMO	H-LGap	*J*(*I*)	*J*(*a*)	*J*(*HL*)	ΔSL
Theopapuamide A	−6.3114	−0.8210	−0.8169	5.4904	0.041	0.000	0.041	0.004
Theopapuamide B	−6.2907	−1.0710	−1.0675	5.2197	0.042	0.002	0.043	0.004
Theopapuamide C	−6.3125	−0.8893	−0.8838	5.4232	0.042	0.000	0.042	0.005
Theopapuamide D	−6.6760	−0.9040	−0.8879	5.7721	0.003	0.010	0.011	0.016

**Table 4:** Global reactivity descriptors for the marine cyclopeptides theopapuamides A-D: electronegativity (χ), hardness (η), electrophilicity (ω) (all in eV), Softness (S) (in eV^−1^), Nucleophilicity (N), electrodonating power ($\omega^{-}$), electroaccepting power ($\omega^{+}$), and net electrophilicity ($\Delta \omega^{\pm }$) (also in eV).

	χ	η	γ	S	N	$\omega^{-}$	$\omega^{+}$	$\Delta \omega^{\pm }$
Theopapuamide A	3.5662	5.4904	1.1582	0.1808	2.8098	4.4426	0.8764	5.3190
Theopapuamide B	3.6809	5.2197	1.2979	0.1900	2.8305	4.7624	1.0815	5.8439
Theopapuamide C	3.6009	5.4232	1.1954	0.1830	2.8087	4.5303	0.9294	5.4597
Theopapuamide D	3.7900	5.7721	1.2443	0.2012	3.2174	4.7443	0.9543	5.6986

## Abbreviations

The following abbreviations are used in this manuscript:

DFT	Density Functional Theory
HOMO	Highest Occupied Molecular Orbital
LUMO	Lowest Unoccupied Molecular Orbital
SOMO	Single Occupied Molecular Orbital
KID	Koopmans In DFT
SMD	Solvation Model Density
QSAR	Quantitative Structure Activity Relationships
IEF-PCM	Integral Equation Formalism-Polarized Continuum Model
IP	Ionization Potential energy
EA	Electronic Affinity
DFTBA	Density Functional Tight-Binding Approximation
DD	Dual Descriptor
MP2	Moller-Plesset 2
MP4	Moller-Plesset 4
CCSD	Coupled Cluster Singles-Doubles

## Appendix A

**Table A1:** Condensed local reactivity descriptors for theopapuamide A denoting the reactivity sites: electrophilic Fukui function $f^{-}$, nucleophilic Fukui function $f^{+}$, radical Fukui function $f^{0}$, electrophilic Parr function P−, and nucleophilic Parr function P+.

	$f^{-}$	$f^{+}$	$f^{0}$	P−	P+
Theopapuamide A	N37 (0.41)	C78 (0.11)	N37 (0.20)	N37 (0.78)	C78 (0.23)

**Table A2:** Condensed local reactivity descriptors for theopapuamide B denoting the reactivity sites: electrophilic Fukui function $f^{-}$, nucleophilic Fukui function $f^{+}$, radical Fukui function $f^{0}$, electrophilic Parr function P−, and nucleophilic Parr function P+.

	$f^{-}$	$f^{+}$	$f^{0}$	P−	P+
Theopapuamide B	N38 (0.41)	C73 (0.09)	N38 (0.20)	N38 (0.78)	C73 (0.19)

**Table A3:** Condensed local reactivity descriptors for theopapuamide C denoting the reactivity sites: electrophilic Fukui function $f^{-}$, nucleophilic Fukui function $f^{+}$, radical Fukui function $f^{0}$, electrophilic Parr function P−, and nucleophilic Parr function P+.

	$f^{-}$	$f^{+}$	$f^{0}$	P−	P+
Theopapuamide C	N37 (0.41)	C78 (0.13)	N37 (0.20)	N37 (0.78)	C78 (0.25)

**Table A4:** Condensed local reactivity descriptors for theopapuamide D denoting the reactivity sites: electrophilic Fukui function $f^{-}$, nucleophilic Fukui function $f^{+}$, radical Fukui function $f^{0}$, electrophilic Parr function P−, and nucleophilic Parr function P+.

	$f^{-}$	$f^{+}$	$f^{0}$	P−	P+
Theopapuamide D	N30 (0.11)	C84 (0.10)	C84 (0.10)	N30 (0.27)	C84 (0.38)
